# Controlled Shape and Porosity of Polymeric Colloids by Photo-Induced Phase Separation

**DOI:** 10.3390/polym11071225

**Published:** 2019-07-23

**Authors:** Elad Hadad, Eitan Edri, Hagay Shpaisman

**Affiliations:** Department of Chemistry, Institute for Nanotechnology and Advanced Materials, Bar-Ilan University, Ramat Gan 5290002, Israel

**Keywords:** photo-induced polymerization, phase separation, polymeric colloids, controlled shape, controlled porosity

## Abstract

The shape and porosity of polymeric colloids are two properties that highly influence their ability to accomplish specific tasks. For micro-sized colloids, the control of both properties was demonstrated by the photo-induced phase separation of droplets of NOA81—a thiol-ene based UV-curable adhesive—mixed with acetone, water, and polyethylene glycol. The continuous phase was perfluoromethyldecalin, which does not promote phase separation prior to UV activation. A profound influence of the polymer concentration on the particle shape was observed. As the photo-induced phase separation is triggered by UV radiation, polymerization drives the extracted solution out of the polymeric matrix. The droplets of the extracted solution coalesce until they form a dimple correlated to the polymer concentration, significantly changing the shape of the formed solid colloids. Moreover, control could be gained over the porosity by varying the UV intensity, which governs the kinetics of the reaction, without changing the chemical composition; the number of nanopores was found to increase significantly at higher intensities.

## 1. Introduction

Control over the shape and porosity of polymeric colloids is a major goal [1], as these two properties have powerful influence over the packing of the colloids [2], deposition upon solvent evaporation [3], and emulsion stabilization [4]. Anisotropic particles can be used for biomedical applications [5,6,7], and the assembly of colloids to colloidal microstructures [8,9,10,11] could be useful for the formation of new functional materials [12]. These particles serve as building blocks for creating complex colloidal structures [13], cubic crystals [14], and staggered linear chains [15]. Various procedures were developed in order to address this goal. Sacanna and Pine [12] surveyed the preparation of polymeric colloids with anisotropic shapes, and outlined the following seven approaches: swelling, phase separation, template-assisted synthesis, controlled deformation, seeded-emulsion polymerization, lithographic design, and particle confinement.

Porosity also has considerable influence on various features of polymeric colloidal systems. In general, materials with pores have higher surface areas than non-porous materials, leading to an increased capacity. This property is crucial for many applications [16], including solid phase extraction [17], chromatography [18], catalytic reactions [19], energy applications [20,21], and drug delivery [22,23]. Common preparation methods rely on the addition of porogens [24]. Porogens are solid, liquid, or gas additives that are incorporated within the desirable matrix and could be later extracted, thus forming pores. Extraction methods include calcination, dissolution by solvents, and exposure to UV light [25]. As an alternative to external porogens, pores can form by phase separation during polymerization. 

The phase separation of polymers in bulk is well studied [26], and occurs when a binary or ternary system in a single phase (a homogeneous one-phase state) is quenched to a two-phased region, also termed the unstable region, which consists of two different phases, usually termed the dispersed and suspended phases, with distinctively different chemical compositions. Researchers have explored [27,28] liquid–liquid phase separation inside emulsion droplets that were formed by microfluidic devices and stabilized with surfactants. While microfluidic devices allow for the formation of droplets with a narrow size distribution on demand, they are limited in the smallest achievable size and are hard to upscale. Fan and coworkers [29] showed the formation of anisotropic Janus particles by phase separation, where control over the shape is achieved by varying the polymer blend. In this study, we propose a novel concept that allows for control over both the shape and porosity of droplets by controlled phase separation induced by photopolymerization. We first introduce the procedure, and then demonstrate how a profound influence on the shape is achieved by varying the polymer concentration. We then show how changing the UV intensity at a fixed chemical concentration effects the porosity because of changes in the polymerization dynamics. Finally, we quantify the changes in the pore distribution.

## 2. Materials and Methods

### 2.1. Materials

Norland Optical Adhesive 81 (NOA81) was purchased from Norland Products (Cranbury, NJ, USA), and used as received. This adhesive was reported as a mixture of mercapto-esters (50–70 wt %) and triallyl isocyanurate (30–50 wt %). Perfluoromethyldecalin (85% pure) was purchased from ABCR (Karlsruhe, Germany), acetone (99.8% pure) was purchased from Daejung (Seoul, South Korea), and polyethylene glycol (PEG) with an average molecular weight of 4000 g/mol was purchased from Fisher Chemical (Loughborough, England). All of the chemicals were used without further purification. De-ionized water (~18 MΩ) was provided from an inhouse source.

### 2.2. Dispersed Phase-NOA81-Mixture-Preparation

A thiol-ene-based UV curable adhesive (NOA81) was mixed with acetone, PEG-4000, and water. NOA81 contains a mixture of mercapto-esters (50%–65%) and triallyl isocyanuarte (30%–55%). The polymerization mechanism upon UV irradiation is illustrated in Figure S3 in the Supplementary Materials. First, a 1:2 weight mixture of PEG and water was prepared. The PEG/water mixture was then mixed with acetone at a 1:4 weight ratio, thus obtaining a 1:2:12 weight ratio of PEG, water, and acetone, respectively. The PEG/water/acetone mixture was added to varying amounts (see below) of the more viscous NOA81 in a 2-ml vial.

### 2.3. Colloidal Formation

Then, 2 µL of the NOA81-mixture was added to a 5 mL vial containing 2 mL of perfluoromethyldecalin oil at 23 °C. A homogenizer (D1000, Benchmark Scientific, Edison, NJ, USA) with a rotor speed of 19,000 rpm was used immediately upon the addition of the dispersed phase. UV irradiation (360 nm; CS2010 by Thorlabs, Newton, NJ, USA) with an intensity of 7–46 mW/cm^2^, measured by a PM16-130 power meter (Thorlabs, Newton, NJ, USA), was applied within 3 s of the beginning of the homogenization process. The combined duration of the homogenization and UV irradiation was 2 or 5 min. A scheme of the polymerization process is shown in Figure S3.

### 2.4. Characterization

For the characterization of the formed colloids, a droplet from the UV-treated perfluoromethyldecalin/NOA81-mixture was deposited on a clean glass cover slide and left for 48 h at ambient conditions, to allow for the evaporation of all of the solvents (perfluoromethyldecalin, acetone, and water). SEM images were obtained using a Quanta FEG 250 System (Thermo Scientific, Hillsboro, OR, USA) at 15 kV. Pore size histograms were obtained by a multidimensional program for analyzing images, Image J.

### 2.5. Cross-Sectioning

The dry NOA81 colloids were transferred into 1-mL Eppendorf safe-lock tubes that were subsequently filled with a PELCO epoxy resin (purchased from TED PELLA, Redding, CA, USA). The epoxy was cured at room temperature for two days in order to fixate the colloids, and was then extracted from the tubes and cross-sectioned by a microtome (PowerTome PC produced by Boeckeler Instruments, Tucson, AZ, USA) with a diamond knife. The slices of epoxy with incorporated colloids were characterized by SEM.

## 3. Results and Discussion

Following the work of Guenthner, Hess, and Cash [30], with some variations, we used a thiol-ene-based UV-curable adhesive (NOA81) mixed with acetone, water, and PEG as a dispersed phase. Acetone is a good solvent for NOA81, while water serves as a non-solvent. PEG is added in order to increase the viscosity [30]. A 1:2:12 weight ratio of PEG-4000, water, and acetone, respectively, was mixed with various weight percentages of NOA81 (10–85 wt %); we refer to the acetone/water/PEG/NOA81 mixture as the “NOA81-mixture” throughout the manuscript.

**Figure 1:**
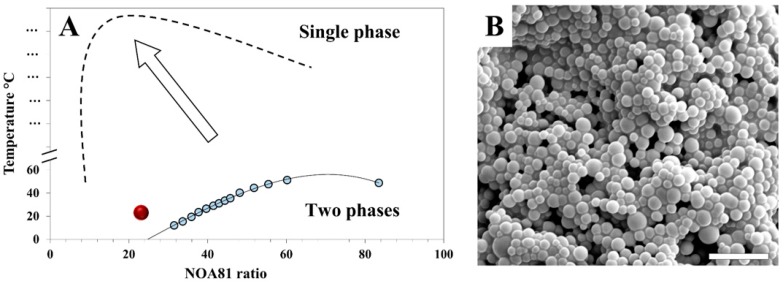
(**A**) Phase diagram of the Norland Optical Adhesive 81 (NOA81)-mixtures before and after UV irradiation. Blue dots are experimental measurements, where a change in turbidity was observed, and construct the binodal curve (solid line). The red dot represents the parameters used for the formation of the structure shown by SEM imaging in (**B**). As the monomers polymerize upon UV irradiation, the binodal curve shifts (dashed line, illustration) and phase separation occurs. Scale bar = 5 µm.

Phase separation can be induced by increasing the molecular weight of the polymer chains during polymerization. A given combination of the polymer ratio and temperature that yields a single-phase system can be driven because of the increase in molecular weight toward a two-phase system. The binodal curve in Figure 1 was found by varying the temperature of the NOA81-mixtures with different ratios. For each ratio, the temperature was found at which the mixture shows a transition from transparent–single-phase to opaque–two-phase. As an example, for phase separation as a result of photo-induced polymerization, we consider a 23 wt % NOA81-mixture at 23 °C (red dot in Figure 1). Prior to UV exposure, the mixture is a transparent liquid (single-phase state); during irradiation, a photochemical reaction instantly takes place, and the monomers polymerize. As a result, the binodal curve shifts considerably (dashed line in Figure 1a), as the polymerized moieties are much less likely to dissolve. Therefore, after UV irradiation, the 23 wt % NOA81-mixture at 23 °C is situated at the two-phase region, and a white opaque solid polymer (Figure 1b) separates from the water/acetone/PEG solvent mixture.

While previous studies of a similar system [30] have demonstrated photo-induced phase separation in bulk, the formation of colloids with controlled morphologies as a result of phase-separation was not shown. In order to form colloids from our dispersed phase (NOA81-mixtures), a fluid that can serve as a dispersant is required. As the mixtures are composed of several components with various properties, it is challenging to find a continuous phase that will not promote phase separation prior to the UV activation of the polymerization. Trials of various carbon oils were performed in order to find a suitable dispersion liquid; however, their addition led to the undesirable separation of one or more components of the NOA81-mixture (see Figure S1 in the Supplementary Materials). Fluorinated carbon oils such as perfluoromethyldecalin could serve as the continuous phase, as they are inert to a wide variety of materials [31,32,33,34,35,36]. They were previously used in microfluidic research, where chemically inert solvents are required [37]. Indeed, the mixing of the NOA81-mixtures with perfluoromethyldecalin did not result in separation (see Figure S1).

**Figure 2:**
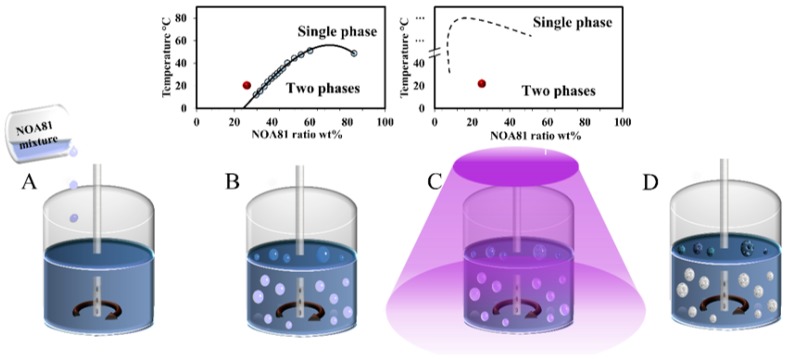
Illustration of colloid formation by photo-induced phase separation of NOA81-mixtures, as follows: (**A**) mixture added to perfluoromethyldecalin oil (blue phase), (**B**) homogenizer forms droplets, (**C**) UV irradiation promotes polymerization and phase separation, and (**D**) solid polymerized NOA81 colloids (white porous spheres) can be extracted.

In order to form colloids, 2 µL of the NOA81-mixture was added to 2 mL of perfluoromethyldecalin oil at 23 °C (Figure 2A). A homogenizer (Figure 2B) and UV source (Figure 2C) were turned on immediately upon the addition of the dispersed phase. The total time of homogenization and UV irradiation was 5 min. The entire system of dispersed, and the suspended liquids can be described as a (W/O)/FO system comprising water (water/acetone) and oil (NOA81) phases in fluorinated oil (FO). Without UV irradiation, the dispersed NOA81-mixture droplets coalesce and cream at the surface of the perfluoromethyldecalin (1.95 g/mL), because of their lower density.

The dispersion droplets formed as a result of homogenization are photopolymerized by UV irradiation, and shift from one-to two-phase state (inset of Figure 2B,C) with the formation of solid colloids (Figure 2D) that were extracted and visualized by SEM. Upon drying, water, acetone, and perfluoromethyldecalin evaporate, yielding polymerized NOA81 colloids.

**Figure 3:**
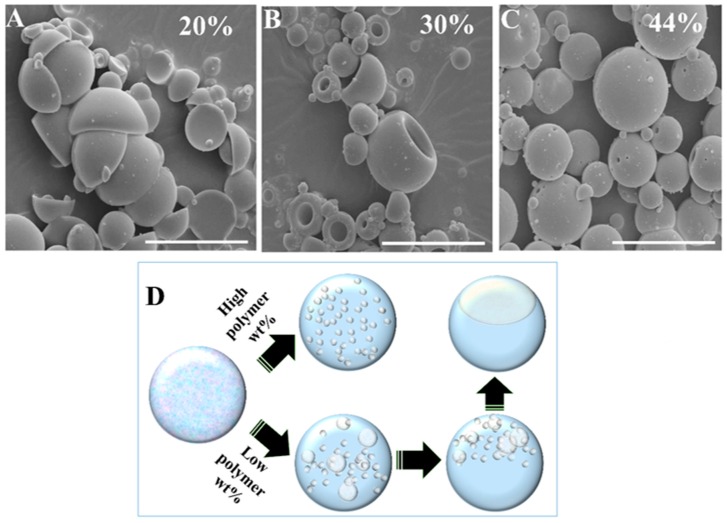
SEM images using a fixed UV intensity (46 mW) demonstrating a change in shape with varying polymer (NOA81) concentrations, as follows: (**A**) 20 wt %, (**B**) 30 wt %, and (**C**) 44 wt %. All of the colloids were dispersed in perfluoromethyldecalin at 23 °C, and irradiated at 46 mW for 5 min. Scale bars = 30 µm. (**D**) Illustration (not to scale) of the formation mechanism of non-spherical colloids using a high UV irradiation intensity: A droplet of the mixed solution starts in a single-phase state. Upon UV irradiation, phase separation occurs. For low polymeric concentrations, the nano-sized droplets of the extracted solution coalesce until they form a dimple. For a high polymeric content, the high viscosity traps the extracted solution, resulting in a spherical colloid.

We observed a profound influence on the shape of the particles as a function of the polymer concentration (achieved by varying the NOA81 weight content). Figure 3 shows the SEM micrographs of the polymeric colloids (NOA81) formed from droplets with a different polymer concentration. All of the colloids were prepared with an irradiation intensity of 46 mW. As is evident from the images in Figure 3A–C, the colloids formed by higher polymer concentrations are more spherical. We attribute this behavior to the manner in which the non-polymeric solution was extracted.

After the phase separation is triggered by UV irradiation, polymerization of NOA81 drives the extracted water/acetone/PEG-4000 solution out of the polymeric matrix. We observed noticeable changes in the colloidal shape as a function of the polymer content using a fixed UV irradiation intensity (46 mW). For relatively high polymer concentrations (44 wt %), the viscosity rises very quickly, trapping the extracted solution (seen as submicron pores, Figure 3C) in almost spherical polymeric colloids. At lower polymer concentrations, the polymeric structure is not rigid enough to maintain a spherical shape. In these cases, as shown in Figure 3A,B for the 20 and 30 wt % polymer, respectively, nano-sized droplets of the extracted solution grow over time as the polymerization progresses inside the forming colloid. Because of the relatively low viscosity for the low polymeric concentrations, the nano-sized droplets of the extracted solution coalesce until they form a dimple (Figure 3D), in order to minimize surface tension. The size of the dimple is correlated with the polymer concentration (a lower polymeric concentration results in bigger dimples), and significant changes to the shape of the formed colloids can be seen in Figure 3A,B for 20 and 30 wt % polymer, respectively. We note that the FTIR measurements did not reveal the presence of PEG in the formed colloids.

**Figure 4:**
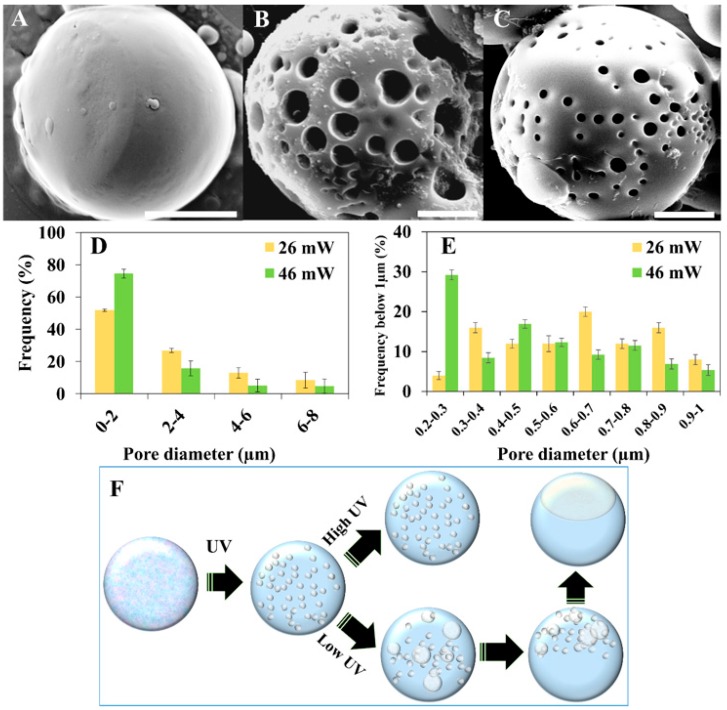
SEM images of the colloids obtained from 38 wt % of a NOA81-mixture at different UV intensities, as follows: (**A**) 8 mW, (**B**) 26 mW, and (**C**) 46 mW. Scale bars = 5 µm. (**D**,**E**) Histograms of the NOA81 colloid pore diameter. (**F**) Illustration (not to scale) of the formation mechanism of non-spherical and porous colloids using a fixed polymer concentration, namely: A droplet of the mixed solution starts in a single-phase state. Upon UV irradiation, phase separation occurs. High UV irradiation intensities leads to high polymerization rates, trapping small droplets of extracted solution in the polymeric matrix. Low intensities allow for more time for the extracted solution to grow and coalesce, resulting in a colloid with a dimple, but almost without pores.

While controlling the shape of the formed colloids is achievable by changing the polymer concentration, as shown above, control over the porosity can also be gained by governing the kinetics of the polymerization, without any change to the chemical composition. We show, in Figure 4, the formation of significantly different colloids, all formed from the same NOA81-mixture (38 wt %), by variation of the UV intensity, which is known to influence the polymerization rate [21]. Applying relatively high UV intensities (46 mW) leads to high polymerization rates, trapping small droplets of extracted solution in the polymeric matrix. This procedure therefore forms spherical colloids with small pores (Figure 4C). Decreasing the UV intensity slows down the polymerization rate, therefore allowing more time for dynamic processes of the extracted solution to occur. When applying 26 mW of UV radiation, droplets of the extracted solution grow significantly, resulting in spherical colloids with larger pores (Figure 4B). Decreasing the UV intensity even further to 8 mW, allows the growing droplets of the extracted solution to coalesce and migrate to the surface of the growing colloid (illustrated in Figure 4F). The resulting colloid is no longer spherical, because of the dimple formed by the extracted solution 

Figure 4A shows how, as the droplets of the extracted solution migrate to the surface of the colloid to form the dimple, the polymerized colloid is no longer porous. Micrographs of additional colloids with various sizes [38] are shown in Figure S2.

Histograms of the pore diameters of the colloids are shown in Figure 4D,E. The pore sizes were determined by analyzing the SEM micrographs of 20 colloids prepared with a UV intensity of either 26 or 46 mW. Figure 4D shows the diameter distribution of the micropores (up to 8 µm), while Figure 4E shows the size distribution of the nanopores. From these histograms, we conclude that using a higher UV intensity during polymerization results in a significant increase in the number of nanopores, matching the mechanism suggested above.

**Figure 5:**
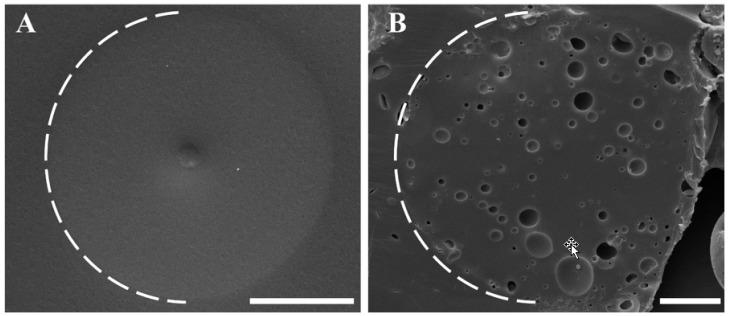
SEM micrographs of colloid cross-sections for a 38 wt % NOA81-mixture formed at different UV intensities, namely: (**A**) 8 mW and (**B**) 26 mW. Scale bars = 15 µm. The white dashed lines show the border between the colloids and the epoxy used to fixate them for cross-sectioning.

In order to understand whether the pores visualized by SEM are only at the surface of the colloids or also inside them, we studied various cross-sections of the colloids, performed by fixation in epoxy and slicing using a microtome. Figure 5A,B shows typical SEM micrographs of cross-sections for colloids prepared with 8 and 26 mW, respectively. The conclusion from these images is that the interior parts of the colloids have the same morphology as their exterior. The colloids that do not show voids at the surface (8 mW) are also free of voids in the interior, while those that seem porous on the outside (26 mW) are also porous on the inside. Moreover, the diameter distribution of the micropores at the colloids’ surface and inside the colloids (as seen after cross sectioning) are similar (see Figure S4 in the Supplementary Materials).

## 4. Conclusions

We demonstrate a novel method that allows for control over the shape and porosity of polymeric micro-sized colloids. This is achieved by controlling the photo-induced phase separation of droplets of a thiol-ene-based UV curable adhesive (NOA81) mixed with acetone, water, and PEG. The continuous phase chosen was perfluoromethyldecalin, which does not promote phase separation prior to UV irradiation. We observed a profound influence on the shape and porosity of the particles as a function of the polymer concentration and UV intensity. This is due to the dynamics by which the solution extracted from the polymerization process coalesces.

## Supplementary Materials

The following are available online at https://www.mdpi.com/2073-4360/11/7/1225/s1, containing the following figures: Figure S1: (**A**) Various hydrocarbon oils at 30 °C (from left to right: octane, cyclohexane, toluene, hexane mixture, benzene, pentane, dichloromethane, ether, heptane, and triethyamine) combined with an NOA81-mixture. In all cases, a turbid phase was obtained as a result of phase separation. (**B**) NOA81-mixture with perfluoromethyldecalin resulting in two distinct clear phases without any turbidity, where (**C**) the upper phase is the NOA81-mixture and the lower phase is perfluoromethyldecalin. Figure S2: SEM images of colloidal particles obtained from a 38 wt % NOA81-mixture at 0 °C following 2 minutes of UV irradiation at different intensities (indicated). Figure S3: Polymerization scheme of mercapto-esters and triallyl isocyanuarte (the components of NOA81) under UV irradiation. Figure S4: Histogram of the colloids’ pores diameter obtained from 38 wt % of NOA81-mixtures using 26 mW of UV intensity.
