# Supplementary material for: Controlled Shape and Porosity of Polymeric Colloids by Photo-Induced Phase Separation
Source: Polymers (Basel). 2019 Jul 23;11(7):1225. doi: 10.3390/polym11071225 (PMC6680483; doi:10.3390/polym11071225)
Supplement: Supplementary file 1 [file polymers-11-01225-s001.pdf]

Supplementary Information

Controlled Shape and Porosity of Polymeric Colloids by Photo-Induced Phase Separation

*Elad Hadad, Eitan Edri and Hagay Shpaisman**

Department of Chemistry, Institute for Nanotechnology and Advanced Materials

Bar-Ilan University, Ramat Gan, 5290002, Israel

*E-mail: hagay.shpaisman@biu.ac.il

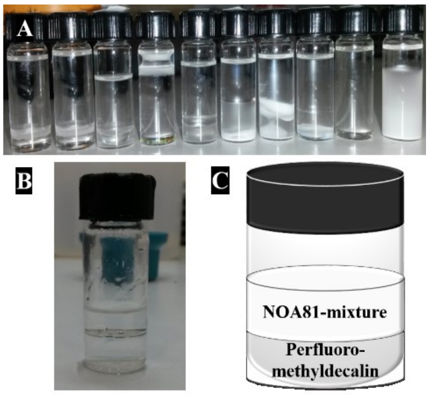

Figure S1. (A) Various hydrocarbon oils at 30 °C (from left to right: octane, cyclohexane, toluene, hexane mixture, benzene, pentane, dichloromethane, ether, heptane and triethyamine) combined with NOA81-mixture. In all cases, a turbid phase was obtained due to phase separation. (B) NOA81-mixture with perfluoromethyldecalin resulting in two distinct clear phases without any turbidity where (C) the upper phase is the NOA81-mixture and the lower phase is perfluoromethyldecalin.

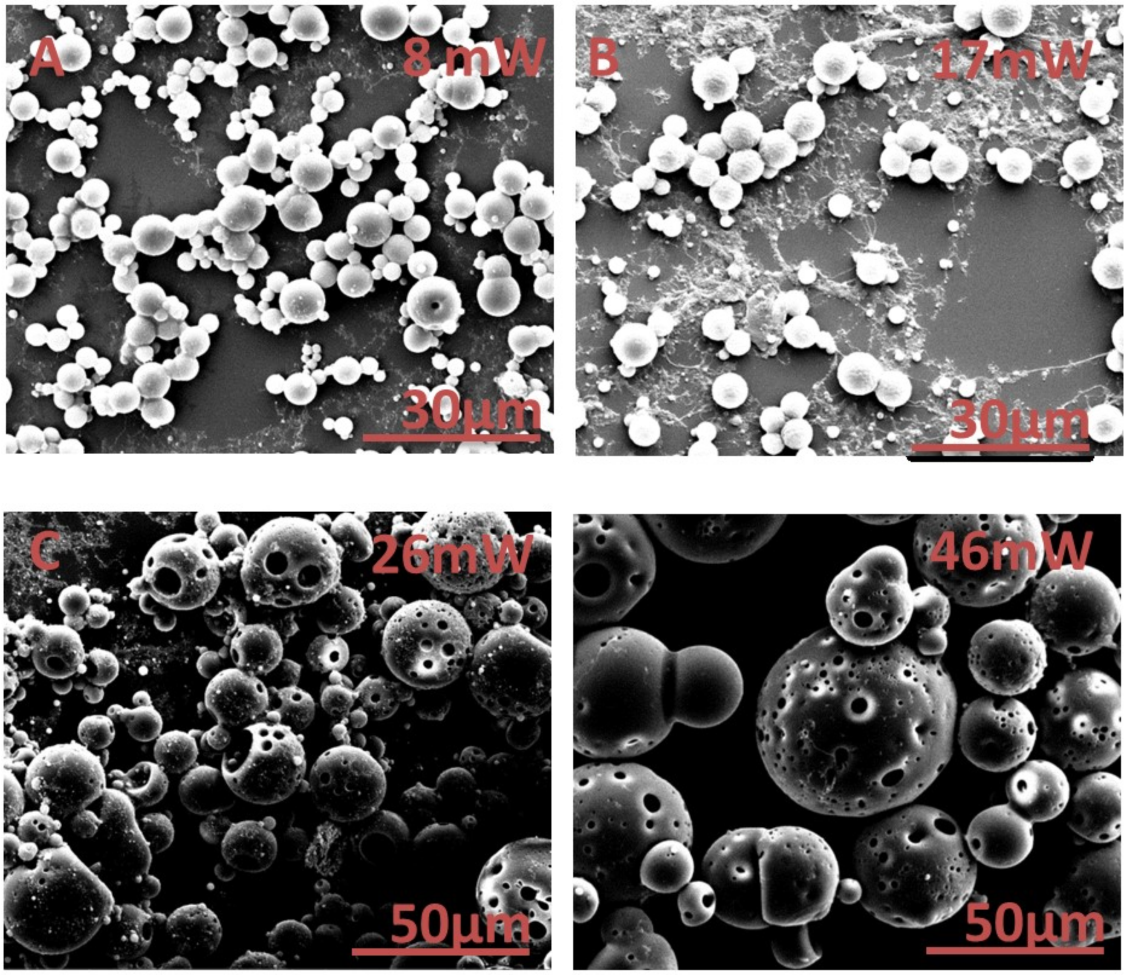

Figure S2. SEM images of colloidal particles obtained from a 38 wt% NOA81-mixture at 0 °C following 2 minutes of UV irradiation at different intensities (indicated).

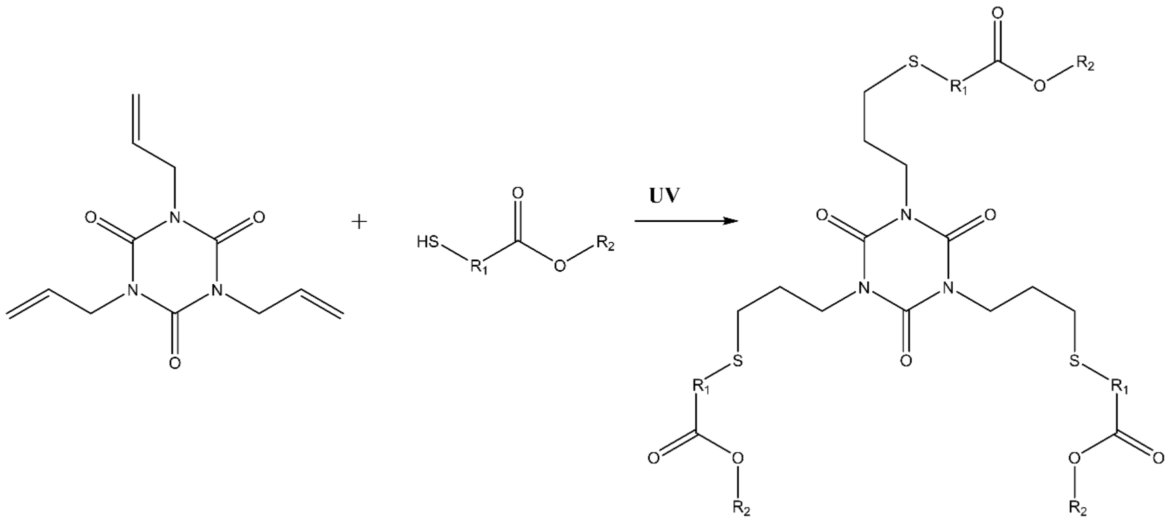

Figure S3. Polymerization scheme of mercapto-esters and triallyl isocyanuarte (the components of NOA81) under UV irradiation.

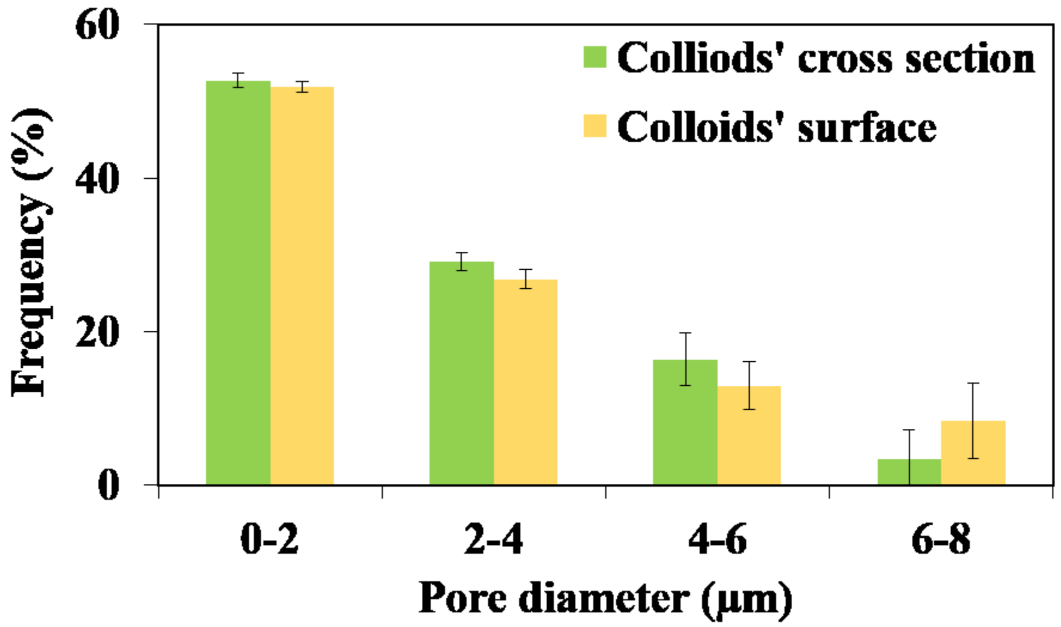

Figure S4. Histogram of the colloids' pores diameter obtained from 38 wt% of NOA81-mixtures using 26 mW of UV intensity.
